# Effects of Environmental Tobacco Smoke on Oxidative Stress in Childhood: A Human Biomonitoring Study

**DOI:** 10.3390/toxics12080557

**Published:** 2024-07-30

**Authors:** Arianna Antonucci, Roberta Andreoli, Chiara Maccari, Matteo Vitali, Carmela Protano

**Affiliations:** 1 Department of Public Health and Infectious Diseases, Sapienza University of Rome, Piazzale Aldo Moro, 5, 00185 Rome, Italy; matteo.vitali@uniroma1.it (M.V.); carmela.protano@uniroma1.it (C.P.); 2 Department of Medicine and Surgery, University of Parma, Via Gramsci 14, 43126 Parma, Italy; roberta.andreoli@unipr.it (R.A.); chiara.maccari@unipr.it (C.M.); 3 Center of Excellence for Toxicological Research (CERT), University of Parma, Via Gramsci 14, 43126 Parma, Italy

**Keywords:** environmental tobacco smoke, children, human biomonitoring, oxidative stress

## Abstract

Household smoking is one of the main sources of environmental tobacco smoke (ETS) exposure for children, a population considered to be at high risk for associated negative health outcomes. Several studies evidenced the occurrence of early effects related to ETS exposure, including the development of the oxidative stress process. The aim of this study was to evaluate the correlation between urinary levels of 8-oxo-7,8-dihydro-2-deoxyguanosine (8oxodGuo), a nucleic acid oxidation biomarker, and socio-demographic features and lifestyle factors in school children (aged 5–11 years). A cross-sectional study was conducted among 154 healthy children, residing in rural zones of central Italy. For each participant, one urine sample was analyzed by the HPLC-MS/MS technique to simultaneously quantify 8oxodGuo and cotinine (a biomarker of ETS exposure), while information on the children was collected using a questionnaire filled out by the parents. Urinary levels of 8oxodGuo was found to be significantly higher in children exposed to ETS compared to those not exposed (5.53 vs. 4.78 μg/L; *p* = 0.019). This result was confirmed by the significant association observed between urinary levels of cotinine and 8oxodGuo (r = 0.364, *p* < 0.0001). Additionally, children exposed to ETS with no smoking ban at home showed a further increased difference than those not exposed (6.35 μg/L vs. 4.78 μg/L; *p* = 0.008). Considering the great number of adverse effects on human health due to exposure to passive smoking, especially if this exposure begins early in life, it is essential to implement health promotion interventions in this area.

## 1. Introduction

Environmental tobacco smoke (ETS) is a well-known risk factor for human health and even short-term exposure can have immediate adverse effects because there is no safe exposure level [1]. Indeed, short-term exposure can cause irritation of the nose, eyes, throat and lungs, headaches, nausea, dizziness, and asthma attacks [2]. Additionally, long-term exposure to ETS results in a significant number of so-called “smoking-related illnesses”, including cardiovascular and respiratory diseases, adverse reproductive outcomes in pregnant women, and various types of cancer [1,2]. ETS contains more than 7000 chemicals, several of which are known or probable carcinogens, toxicants, and inducers of oxidative stress [3], a process closely involved in carcinogenesis and in the development of other chronic diseases [4]. A very recent systematic review reported that both active and passive cigarette smoking induce oxidative stress, even though, at present, there is limited scientific evidence on the effects of ETS on the health of smokers and non-smokers [5].

Children are a group of the population particularly susceptible to the adverse effects of ETS exposure because of their less developed immune systems and smaller lungs compared to adults. Exposure to ETS in children can cause acute and/or chronic respiratory diseases, ear infections, and a reduction in lung function [2]. In the year 2019, it was estimated that ETS exposure was responsible for 4,500,000 disability-adjusted life years and 50,000 deaths among children under 14 years [6]. This is a critical issue for public health because, despite strong scientific evidence on the association between passive smoking exposure and the occurrence of negative outcomes for human health, especially for children, a high percentage of children are exposed worldwide [7].

In order to reduce exposure to ETS, many countries, including Italy, have introduced smoking bans in public areas, but this kind of restriction cannot be applied in domestic environment, which remains a place where passive smoking exposure occurs [8]. In this regard, a recent survey on the status and correlation of children’s exposure to ETS demonstrated that ETS exposure was associated with several factors: the time in which cohabitant smokers smoke their first cigarette of the day, changes in smoking habits within the last six months, the efficacy and self-efficacy to maintain a smoke-free home, and the attitude towards offering cigarettes to guests in the domestic environment [9]. However, it must be remembered that ETS is a combination of secondhand smoke, that is, the smoke emitted by people during smoking or immediately after finishing smoking, and thirdhand smoke, that is, the smoke that persist for weeks on the body, furnishings, clothing, etc., of smokers after cigarette smoking [10]. Thus, individuals living with one or more smokers are continuously exposed to the components of ETS, as demonstrated by several studies previously [11,12,13]. Scientific evidence also showed that exposure to ETS can lead to effects such as the induction of the oxidative stress process, as studied through specific biomarkers, both in adults [14,15,16] and children [17], while in other cases no significant associations were found [18]. These differences may be due in part to the specific smoking behaviors (years of smoking, number of cigarettes smoked per day, smoking indoors, etc.) and the specific level of passive smoking exposure, in part, to the biomarkers used and their analytical determinations. Thus, the effect of active smoking and/or ETS exposure on the oxidative stress process and the association between ETS exposure and biomarkers of oxidative stress should be studied in depth.

The present study aimed to evaluate the influence of ETS exposure at home on oxidative stress using the biomarker 8-oxo-7,8-dihydro-2′-deoxyguanosine (8oxodGuo) among a group of primary school children (5–11 years old).

## 2. Materials and Methods

### 2.1. Study Design

A cross-sectional study was conducted among a group of healthy children attending primary schools situated in a countryside area in central Italy. This study was approved by the local Ethical Committee with the protocol code n. 2894. Anonymous treatment was guaranteed, and all data were processed only for scientific purpose. In total, three primary schools in Rieti province were invited to participate in the study. The features of the selected areas and precise procedures to participate in the biomonitoring study (registration of children and collection of data and urine samples of participants) were previously described [11,12]. The sampling zone was far from high-traffic roads, and the subjects (aged 5 to 11 years) were recruited on a voluntary basis, after having received, together with their parents, the complete information on the design and purpose of the project. Based on the number of children attending the selected schools, the research team prepared one full set of material for each potential participant (operating guide, informed consent, personal data processing authorization, self-administered questionnaire, polypropylene bottle for urine collection, and adhesive code label) and brought the kits to the schools taking part in the project. Then, the teachers delivered the personal kits to their students.

### 2.2. Information Gathered by Questionnaire

The questionnaire was specifically designed to collect data on the socio-demographic characteristics, living conditions, the habits of smokers living with the child, and daily activities. In particular, we investigated the following categories: gender, ponderal status (grouped as “underweight and normal weight” or “overweight and obese”, according to sex-specific body mass index for age growth charts produced by the Centers for Disease Control and Prevention) [19], educational level of each parent (basic: ≤9 years, upper secondary: ≤14 years or tertiary/higher: ≥17 years, according to the Organization for Economic Cooperation and Development) [20,21], house density measured as square footage × inhabitant (high density: ≤30 m^2^/inhab. or low density: >30 m^2^/inhab) [12], extra-curricular activities (no activity or at least one daily activity), and ETS exposure status in the domestic environment (no or yes, when at least one cohabitant smoker is present). Then, in order to explore the influence of domestic ETS exposure on cotinine and 8oxodGuo urinary levels, only in children exposed to ETS, we considered the following variables: home smoking policy (total ban: cohabitant smoker(s) do not smoke in the domestic environment; partial ban: cohabitant smoker(s) smoke in the domestic environment just when the child is not present; no ban: cohabitant smoker(s) smoke in the domestic environment without any prohibition), number of cohabitant smoker(s) (one smoker; >one smoker), total cigarettes consumed each day (≤20 cigarettes, >20 cigarettes), and cigarettes consumed in the domestic environment (≤10 cigarettes, >10 cigarettes).

Questionnaires were filled by the adult caretakers of the child. All data collected from questionnaire replies were coded and entered in a database specifically constructed for statistical purpose. Details on assignment of related codes were previously reported [22].

### 2.3. Urine Sample Collection

For each participant, one spot urine sample was collected in the evening (just before bedtime). Urine samples were collected in the evening because both cotinine and 8oxodGuo have short biological half-lives in the human body [23,24]. Thus, the concentration of cotinine and 8oxodGuo in the evening samples reflects the exposure and consequent effects that occurred during the preceding hours, when most participants spent their time at home and, if they lived with cohabitant smoker(s), were most likely exposed to ETS. After the collection, urine samples were stored in a domestic refrigerator and delivered to school in the morning of the day after, along with the completed questionnaire and other documentation. In the morning, all materials were transported to the laboratory by the research team and each spot sample was separated into aliquots, labeled with an alphanumeric ID, and stored in freezer at −20 °C until analyses. The first aliquot was evaluated for the urinary concentration of both biomarkers of oxidative stress (8oxodGuo) and exposure to ETS (cotinine). Additionally, an additional aliquot was used to determine creatinine level.

### 2.4. Analytes Determination

Urinary determinations were carried out through isotopic dilution liquid chromatography–tandem mass spectrometry (LC–MS–MS) using an ABSCIEX API 4000 triple-quadrupole mass spectrometer (AB SCIEX, Framingham, MA, USA) equipped with a TurboIonSpray interface for pneumatically assisted electrospray (TIS). Analyses were performed in a laboratory that, in the past, was involved in an inter-laboratory project, which included quality control for assessing the urinary level of 8oxodGuo, organized by the European Standards Committee on Urinary (DNA) Lesion Analysis. Each year different aliquots of urine were spiked with two different levels of 8oxodGuo and cotinine, which were used as quality controls (low and high) for each analytical session. Details on the procedure were reported previously [25]. Urinary-free cotinine and 8oxodGuo were analyzed in the same chromatographic run, according to the method reported in detail in a previous study by Andreoli et al. [26] with minor modifications. Briefly, after thawing, an urinary aliquot (0.2 mL) was vortexed and centrifuged (10 min at 10,000× *g*); then, 50 μL of supernatant was added with 150 μL of ISs aqueous mixture containing [15N5]8oxodGuo (2.5 μg/L, self-made in lab starting from [15N5]dGuo, Cambridge Isotope Laboratories, Inc., Tewksbury, MA, USA) and cotinine-d3 (200 μg/L, Sigma Aldrich, Milan, Italy) and injected (2 μL). Chromatography was performed with an Atlantis^®^dC18 column (100 mm × 2.0 mm i.d., 3 μm; Waters, Milford, MA, USA) using variable proportions of 10 mM aqueous formic acid (pH = 3.75) and methanol from Sigma Aldrich (Milan, Italy) at a flow rate of 0.2 mL/min. The stock solution (about 1 g/L) of 8oxodGuo was prepared in dimethyl sulfoxide (Sigma Aldrich, Milan, Italy). The LODs, calculated as the ratio signal/noise > 3, were 0.05 and 0.15 μg/L for 8oxodGuo and cotinine, respectively, and LOQs calculated as the ratio signal/noise > 10 were 0.2 and 0.5 μg/L. The urinary concentrations of 8oxodGuo and cotinine were always higher than the LOQs. The %CV (at 1 μg/L) was in the range of 2.0% and 6.8% for the two studied compounds and for all intra- and inter-day determinations. Based on WHO’s quality assurance guidelines for sample exclusion [27], we used the recommendation on the urinary creatinine levels of American Conference of Governmental Industrial Hygienists [28] as exclusion criteria: very diluted or very concentrated urine samples (creatinine concentration lower than 0.3 g/L or higher than 3.0 g/L, respectively) were excluded. Creatinine concentrations were obtained by the method of Jaffe [29].

### 2.5. Statistics

The SPSS Statistics software package (25.0 version for Windows, IBM Corp., Armonk, NY, USA) was used to perform statistical analyses. First of all, we reported absolute and relative frequencies for categorical variables and the arithmetic mean ± SD for urinary 8oxodGuo. Urinary cotinine and 8oxodGuo levels followed a log-normal distribution, as assessed by the one-sample Kolmogorov–Smirnov test. Thus, the parametric statistical tests were applied to natural log-transformed values. In particular, the parametric *t*-test for independent variables was used to evaluate differences between two groups and one-way analysis of variance (ANOVA) with Bonferroni post hoc test was used for comparisons among more than two groups. Spearman’s correlation was used for assessing relationships between urinary biomarker levels (8oxodGuo, cotinine and creatinine). Missing values were treated using pairwise deletion. No outlier values were found using the z-score method. Finally, multiple linear regression analysis was performed to evaluate the independent role of some selected possible predictors on the urinary concentration of 8oxodGuo. A significance level of 0.05 (two-tailed) was used for all tests.

## 3. Results and Discussion

In total, 179 healthy children (about the 70% of study population) participated in the current biomonitoring study. Each participant was asked to provide the completed questionnaire and one urine sample to take part in this study. Then, given the possible influence of ethnicity on biomarker concentrations, only children of Caucasian origin [20,30,31] were included in the study and, among these, only children providing an adequate urine sample were selected. Finally, 154 samples, all providing detectable (higher than LOQ) urinary biomarker of nucleic acid oxidation and cotinine, were available for statistical processing. Table 1 reports the main features of the participants and the arithmetic mean ± SD of corresponding urinary 8oxodGuo (μg/L). *p* values, estimated on ln-transformed urinary concentrations, were also reported.

The average age of participants was 8.84 (not reported in Table 1). Among the studied children, a slight majority of whom were male (54.6%), only about 50% had a healthy weight. Although this result does not concern the main objective of our study, it cannot be overlooked from a public health perspective. In particular, almost a third of the participants were overweight or obese, which is in line with the results of research carried out in the same area [21] and with findings observed in children living in other countries [32,33]. This is of great concern because currently childhood overweight and obesity are two of the most important factors affecting public health globally due to their physical, psychological, and negative social consequences [34].

Most of the participants (61.0%) lived in high-density homes (≤30 m^2^/inhabitants) and did not practice any physical activities during the sampling day (56.5%). Finally, a considerable percentage of the participants was exposed to ETS (48.7%). The data on ETS exposure are similar to those reported by a previous study conducted in the same area more than a decade ago [11], thus evidencing that efforts made in recent years to reduce this type of exposure, especially in susceptible subjects such as children, have been unsuccessful.

Considering the investigated biomarker, urinary concentrations were higher for female, overweight, and obese children, children not practicing any sport activity in the day of sampling, children living in a house characterized by square footage × inhabitant ≤ 30 m^2^/inhabitants, and children with parents with basic school education; however, all these results were not statistically significant.

With regard to ETS exposure, data reported by the questionnaires were supported by the urinary levels of cotinine of children not exposed and exposed to ETS, 2.20 and 6.74 μg/L (*p* < 0.0001), respectively.

The results of statistical analyses carried out for assessing the association between ETS exposure and the urinary levels of the oxidative stress biomarker demonstrated a significantly higher level of 8oxodGuo in urine of ETS exposed compared to the group not exposed to ETS (*p* = 0.019).

**Figure 1:**
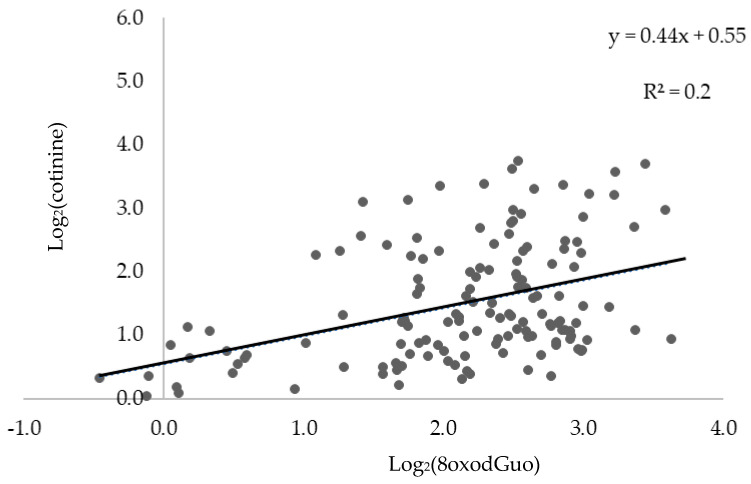
Correlation between the two investigated biomarkers: Log_2_(cotinine) (biomarker of exposure) and Log_2_(8oxodGuo) (biomarker of effect).

The scatter plot of log_2_-transformed u-levels of cotinine and 8oxodGuo (Figure 1) indicate a weak positive linear relationship between the two biomarkers. The correlation would be improved by increasing the number of samples.

Spearman’s correlation coefficients obtained when considering all participants confirmed a statistically significant positive correlation between the urinary levels of 8oxodGuo and cotinine (r = 0.364, *p* < 0.0001) and creatinine (r = 0.450, *p* < 0.0001). These findings are in contrast with the results reported previously that evidenced no significant association between oxidative stress biomarkers and exposure to passive smoking in children [18,35]. These differences could be explained partially by the behavior of the biomarkers and partially by the smoking habits of the cohabitant smokers. With regard to the first point, repair mechanisms of nucleic acid and nucleobase could reduce the pro-oxidative effects of smoking exposure among the general population [36,37]. This effect can be attributed to the ability of individual endogenous and exogenous antioxidants to function as a free radical defense mechanism. Indeed, every human contains enzymes that can protect the cells during the metabolism of oxygen, such as superoxide dismutase, catalase, glutathione systems, and glucose-6-phosphate dehydrogenase. Additionally, subjects can be assumed to have exogenous antioxidants like vitamins, selenium, phenolic compounds, curcumin, tannins, and others [38]. In addition, differences in the urinary excretion of 8oxodGuo could be attributed to the specific uptake of oxidizing agents, including during cigarette smoking, which is influenced by the specific habits of the cohabitant smokers (habit of smoking indoor, number of cigarettes smoked each day, etc.) just like the individual level of ETS exposure of the subject exposed to passive smoking (for example, the time spent in an indoor environment in which others smoke). In light of these findings, some variables characterizing the exposed population were analyzed. Table 2 reports the characteristics of children exposed to ETS and the concentrations of investigated urinary biomarkers (8oxodGuo and cotinine, expressed as μg/L) based on the smoking habits of the cohabitant smoker(s) in the domestic environment.

The urinary levels of 8oxodGuo enhanced slightly, but not significantly, with the number of cohabitant smokers (5.51 μg/L for 1 cohabitant smoker vs. 5.65 μg/L for more than one cohabitant smoker) and the number of the cigarettes smoked daily (5.17 μg/L up to 10 cigarettes vs. 5.60 μg/L for more than 10 cigarettes). The results obtained regarding the number of cigarettes consumed by cohabitant smokers are in line with the finding reported by Yildirim et al. [39], where a significant association between the number of cigarettes consumed by cohabitants and oxidant status of children exposed to passive smoking was found.

On the other hand, data on home smoking policy are not clear because no gradual increase in urinary biomarker concentrations was observed, as would be expected when moving from total ban to no ban on smoking in the domestic environment (5.40 μg/L, 4.21 μg/L, and 6.35 μg/L for total, partial, and no home smoking ban, respectively). Nevertheless, the cohabitant’s habit to smoke at home in presence of children was associated with the highest level of the effect of the investigated biomarker (6.35 μg/L). In order to obtain further information about the influence of home smoking policy on the urinary level trends, we compared the urinary levels of children whose parents smoked at home (without distinguishing between total and partial permission) with children not exposed to ETS. Then, for all children whose cohabitants smoked at home, levels of the investigated biomarker were significantly higher (4.78 μg/L vs. 5.73 μg/L, *p* = 0.022), even when we do not consider the presence of the child at home. If we then exclusively select children exposed to ETS with no smoking ban at home, the difference with children not exposed to ETS further increased (4.78 μg/L vs. 6.35 μg/L, *p* = 0.008). These results proved that the adopted measures of smoking policy in household environments where children live could determine the occurrence of the oxidative stress biomarker process. On the other hand, total smoking ban seems not to protect children from ETS exposure, as children living in homes with partial smoking ban had lower concentrations of urinary 8oxodGuo compared to those obtained under total smoking ban. In this case, other factors, such as the number of cigarettes smoked daily, could more effectively influence the results. Nevertheless, exposure to direct smoke (SHS) in homes with no smoking ban seems to be a crucial factor inducing the oxidative stress process in pediatric population.

Finally, we performed a multivariate analysis to assess the independent role of gender, age, urinary creatinine levels, and ETS exposure on 8oxodGuo concentrations (Table 3).

Significant predictors of urinary 8oxodGuo excretion were exposure to ETS (β-coefficient = 0.169; *p*-value = 0.008) and urinary creatinine levels (β-coefficient = 0.546; *p*-value < 0.001). These regression models explained 57.7% of the variance in urinary levels of 8oxodGuo. This result suggests that other variables (e.g., genetic polymorphisms, food intake, etc.) can impact the urinary excretion of the investigated biomarker. These potential predictors should be studied further in depth.

The present study has some limitations. Firstly, it is a cross-sectional study and, thus, it does not allow us to evidence a causal relationship between the investigated variables. Secondly, the sample size is not large; however, although this is a major limitation of the present work, studies on children are very limited and, thus, the results reported here are of importance. Finally, we did not consider variables such as genetic polymorphisms, food intake, and others. Indeed, the small difference in urinary levels of 8oxodGuo between ETS-exposed and ETS-not-exposed children (5.53 μg/L vs. 4.78 μg/L) suggests that there are multiple sources of oxidative stress among children. Regarding genetic polymorphisms, it is known that cytochrome P450 2A6 (CYP2A6) catalyzes the metabolism of nicotine and the tobacco-specific carcinogens [40]. Thus, genetic variation in CYP2A6 may influence the metabolism of nicotine and cotinine and, consequently, the urinary levels of cotinine. However, scientific evidence demonstrated that CYP2A6 genotypes were not associated with ETS exposure in adults [41]. Future studies should also confirm the lack of association in childhood. With regard to the influence of food intake, dietary pattern including foods rich in polyphenols, polyunsaturated fatty acids, and fiber positively regulated oxidative stress in adult population [40,42]. Also, in this case, the contribution of dietary intake on oxidative stress should be studied in children.

Further research with a high number of participants and considering other potential interfering/confounding factors should be performed to increase the robustness of evidence in this field.

## 4. Conclusions

The results of this study evidenced that passive smoking exposure and some smoking habits of cohabitant smoker(s) caused an increase in urinary concentration of 8oxodGuo, a biomarker of oxidative stress, in childhood. As oxidative stress biomarkers are early effect biomarkers, they can be successfully used to detect tobacco smoke-related diseases in the very initial phase of development. Further research is needed to clarify the role of ETS exposure on the oxidative stress process among children and to identify major potential interfering/confounding factors.

## Figures and Tables

**Table 1:** Main features of participants. U-cotinine mean concentrations (μg/L) were reported according to the children’s ETS exposure status. *p* values were reported for ln-transformed values and marked by an asterisk when referring to statistically significant differences.

		Descriptives in % If Not Stated Otherwise (*n*)	[8oxodGuo] μg/L AM (SD) ^1^	*p* ^2^
Variable		Rieti Province (*n* = 154)		
Age	5–8	44.1 (*n* = 68)	5.05 (2.16)	0.991
	9–11	55.9 (*n* = 86)	5.22 (2.42)	
Gender	male	54.6 (*n* = 84)	4.95 (2.30)	0.255
	female	45.4 (*n* = 70)	5.38 (2.31)	
Ponderal status according to body mass index	Under/Normal weight	55.2 (*n* = 85)	4.86 (2.31)	0.084
	Over/obesity	24.0 (*n* = 37)	5.30 (2.13)	
	Unknown	20.8 (*n* = 32)	5.73 (2.42)	
Maternal education (years)	Basic (≤9 years)	2.6 (*n* = 4)	5.37 (3.46)	0.990
	Secondary (≤14 years)	84.4 (*n* = 130)	5.15 (2.36)	
	Tertiary (≥17 years)	10.4 (*n* = 16)	4.94 (1.91)	
	Unknown	2.6 (*n* = 4)	5.51 (0.89)	
Paternal education (years)	Basic (≤9 years)	2.6 (*n* = 4)	5.73 (3.32)	0.749
	Upper secondary (≤14 years)	85.8 (*n* = 132)	5.24 (2.32)	
	Tertiary/higher (≥17 years)	5.8 (*n* = 9)	4.72 (2.10)	
	Unknown	5.8 (*n* = 9)	4.93 (2.81)	
Home Density (m^2^/inhabitant)	≤30	61.0 (*n* = 94)	5.30 (2.37)	0.565
	>30	31.2 (*n* = 48)	4.92 (2.17)	
	Unknown	7.8 (*n* = 12)	4.90 (2.43)	
Extra-curricular sport activity/day ^3^	No	56.5 (*n* = 87)	5.25 (2.20)	0.421
	Yes	41.6 (*n* = 64)	4.95 (2.63)	
	Unknown	1.9 (*n* = 3)	5.28 (1.41)	
Exposure to ETS ^4^	Not exposed	50.7 (*n* = 78)	4.78 (2.29)	0.019 *
	Exposed	48.7 (*n* = 75)	5.53 (2.29)	
	Unknown	0.6 (*n* = 1)	5.50	
			**[cotinine] μg/L** **AM (SD)** **^1^**	
All participants			4.46 (5.06)	
Exposure to ETS	Not Exposed		2.20 (1.45)	*p* < 0.0001 *
	Exposed		6.74 (5.40)	

^1^ Arithmetic Mean (Standard Deviation); ^2^ *p* estimated on natural log-transformed urinary concentrations; ^3^ the sampling day; ^4^ ETS: environmental tobacco smoke.

**Table 2:** u-8oxodGuo mean concentrations (μg/L) according to home smoking habits of cohabitant smoker(s). *p* values were reported for ln-transformed values and marked by an asterisk when referring to statistically significant differences.

Variable				
		Only Exposed to ETS ^1^ (*n* = 75)	[u-8oxodGuo] μg/L (SD)	*p* ^2^
Number of cohabitant(s) smoker(s)	1	68.0 (*n* = 51)	5.51 (2.31)	0.808
	>1	32.0 (*n* = 24)	5.65 (2.32)	
Cigarettes consumed daily	≤10	17.3 (*n* = 13)	5.17 (2.28)	0.541
	>10	82.7 (*n* = 62)	5.60 (2.31)	
Home Smoking Policy	Never at home	53.3 (*n* = 40)	5.40 (2.49)	0.428
	Only when child is out	12.0 (*n* = 9)	4.21 (1.71)	
	Even in presence of child	29.3 (*n* = 22)	6.35 (2.33)	
	Unknown	5.4 (*n* = 4)	5.28 (2.28)	
Smoking at home	Partial and no home smoking ban ^3^		5.73 (2.32)	0.022 * ^4^
	Smoking at home in presence of child ^5^		6.35 (2.33)	0.008 * ^4^

^1^ ETS: environmental tobacco smoke; ^2^ *p* estimated on natural log-transformed urinary concentrations; ^3^ not considering the presence of children; ^4^ matching with children not exposed to ETS; ^5^ no smoking ban.

**Table 3:** Significant predictors of the urinary levels of 8oxodGuo (µg/L).

Independent Variable	B (Regression Coefficient)	Standard Error	β (Regression Standardized Coefficient)	*p*-Value	Adjusted R^2^
Constant	0.675	0.250	-	0.008	
ETS exposure status	0.151	0.068	0.169	0.027	0.577
Urinary creatinine	0.722	0.100	0.546	<0.001	

Variables included in the multiple linear regression models (forward method): gender (female vs. male), age (as continuous variable), urinary creatinine (u-creatinine as a continuous variable), and ETS exposure status (no vs. yes).

## Data Availability

Data are provided in tables and figures directly within the manuscript, and raw data are available via e-mail upon request to the corresponding author.
